# Ruminal *Prevotella* spp. May Play an Important Role in the Conversion of Plant Lignans into Human Health Beneficial Antioxidants

**DOI:** 10.1371/journal.pone.0087949

**Published:** 2014-04-07

**Authors:** Ana L. B. Schogor, Sharon A. Huws, Geraldo T. D. Santos, Nigel D. Scollan, Barbara D. Hauck, Ana L. Winters, Eun J. Kim, Hélène V. Petit

**Affiliations:** 1 Institute of Biological, Environmental, and Rural Sciences (IBERS), Aberystwyth University, Aberystwyth, United Kingdom; 2 Departamento de Zootecnia, Universidade Estadual de Maringá, Maringá, PR, Brazil; 3 Department of Animal Science, Kyungpook National University, Sangju, Korea; 4 Dairy and Swine Research and Development Centre, Agriculture and Agri-Food Canada, Sherbrooke, QC, Canada; Charité, Campus Benjamin Franklin, Germany

## Abstract

Secoisolariciresinol diglucoside (SDG), the most abundant lignan in flaxseed, is metabolized by the ruminal microbiota into enterolignans, which are strong antioxidants. Enterolactone (EL), the main mammalian enterolignan produced in the rumen, is transferred into physiological fluids, with potentially human health benefits with respect to menopausal symptoms, hormone-dependent cancers, cardiovascular diseases, osteoporosis and diabetes. However, no information exists to our knowledge on bacterial taxa that play a role in converting plant lignans into EL in ruminants. In order to investigate this, eight rumen cannulated cows were used in a double 4×4 Latin square design and fed with four treatments: control with no flax meal (FM), or 5%, 10% and 15% FM (on a dry matter basis). Concentration of EL in the rumen increased linearly with increasing FM inclusion. Total rumen bacterial 16S rRNA concentration obtained using Q-PCR did not differ among treatments. PCR-T-RFLP based dendrograms revealed no global clustering based on diet indicating between animal variation. PCR-DGGE showed a clustering by diet effect within four cows that had similar basal ruminal microbiota. DNA extracted from bands present following feeding 15% FM and absent with no FM supplementation were sequenced and it showed that many genera, in particular *Prevotella* spp., contributed to the metabolism of lignans. A subsequent *in vitro* study using selected pure cultures of ruminal bacteria incubated with SDG indicated that 11 ruminal bacteria were able to convert SDG into secoisolariciresinol (SECO), with *Prevotella* spp. being the main converters. These data suggest that *Prevotella* spp. is one genus playing an important role in the conversion of plant lignans to human health beneficial antioxidants in the rumen.

## Introduction

Several human studies have revealed that ingestion of plant lignans, which are polyphenolic compounds classified as phytoestrogens, can decrease the incidence of menopausal symptoms, hormone-dependent cancers, cardiovascular diseases, osteoporosis and diabetes [1]–[5]. Flax (*Linum usitatissimum*) is the richest source of lignans [1], with secoisolariciresinol diglucoside (SDG) representing more than 95% of all flax lignans. Lignans are mainly found in the fibre portion of flax [6], thus resulting in higher concentration of lignans in hulls than seeds [7].

**Figure 1:**
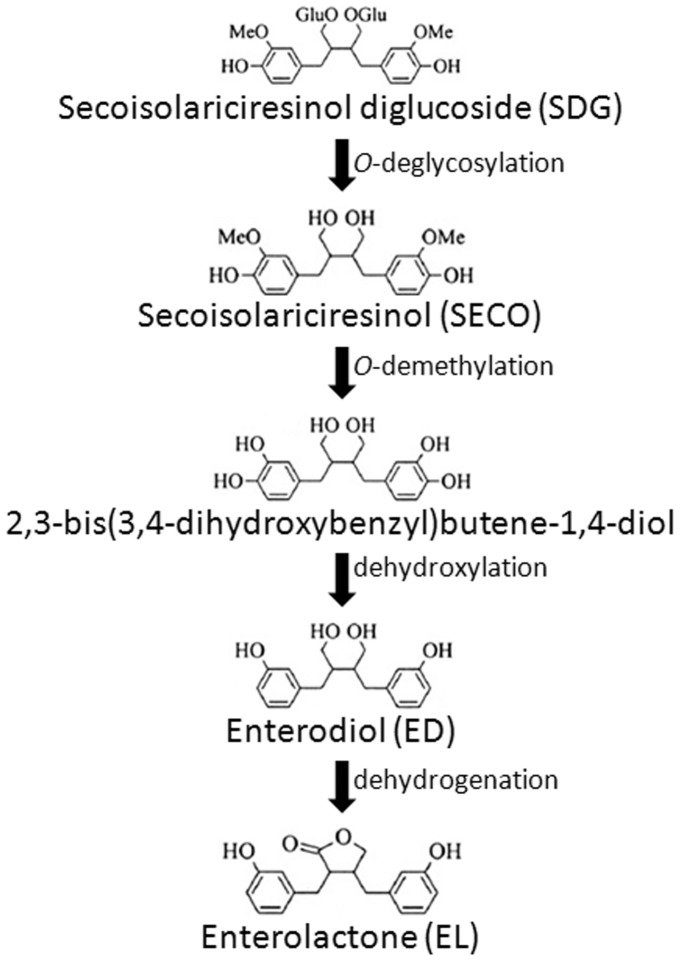
Pathway of enterolignans production from SDG by human faecal bacteria (Adapted from [8]).

In monogastric animals, SDG is converted into secoisolariciresinol (SECO) under the action of intestinal glycosidases and the colonic microbiota convert SECO to the mammalian lignans enterodiol (ED) and enterolactone (EL) [8], [9]. The beneficial effects of flax lignans on human health is due to their antioxidant activity as SECO, ED, EL, and SDG are 4.86, 5.02, 4.35, and 1.27 times more potent than vitamin E as an antioxidant [2]. The conversion of plant SDG into mammalian lignans in humans is basically described by four catalytic reactions: *O-*deglycosylation, *O-*demethylation, dehydrogenation and dehydroxylation [10], [11] (Fig. 1). Some of the human intestinal bacteria involved in the catalytic reactions are strains of *Klebsiella* [12], *Bacteroides distasonis*, *B. fragilis*, *B. ovatus*, *Clostridium cocleatum*, *Clostridium* sp. SDG-Mt85-3Db, *Butyribacterium methylotrophicum*, *Eubacterium callendari*, *E. limosum*, *Peptostreptococcus productus*, *C. scindens*, *Ruminococcus productus*, *Eggerthella lenta* and ED-Mt61/PYG-s6 [8], [10], [13], [14].

Previous studies [7], [15]–[17] have demonstrated that the ruminal microbiota metabolize flax lignans mainly into EL, akin to the situation in the human gastrointestinal tract. Later studies have shown that the metabolism of flax lignans occurs mainly in the rumen and not in the intestine [18] and that EL is the major mammalian lignan in milk [19]. The EL is present in urine, blood, and milk, indicating that phytoestrogens can be transferred to physiological fluids [18]. Indeed, EL concentration in milk increases by feeding flax products, with higher concentrations observed with 15% inclusion of FM in the diet [16], [20], [21]. Since EL has shown antioxidant activity [2], high levels of EL in milk may prevent oxidation [22] and increase shelf life of milk and dairy products.

The rumen microbiota responsible for the conversion of flax lignan into mammalian lignans is nonetheless unknown. Therefore, the aims of this study were to evaluate the effects of inclusion of FM in the diet of dairy cows on the ruminal microbiota using terminal restriction fragment length polymorphisms (T-RFLP), denaturing gradient gel eletrophoresis (PCR-DGGE) and quantitative polymerase chain reaction (Q-PCR). DGGE bands potentially linked with SDG conversion to SECO, ED and EL were sequenced. Subsequently, pure culture studies using genera indicated from the sequences obtained by DGGE as being involved in the process, coupled with high pressure liquid chromatography (HPLC) for detection of antioxidants originating from SDG conversion were completed to ascertain their potential role in antioxidant production in the rumen. Thus, ultimately this study aimed to further our understanding of antioxidant production from SDG by identifying the ruminal bacteria that potentially play a role in this process.

## Materials and Methods

### Animals and treatments

All experimental procedures were approved by the local Animal Care Committee of the Dairy and Swine Research and Development Centre from Agriculture and Agri-Food Canada, Sherbrooke, Canada. Cows were cared for in accordance with the guidelines of the Canadian Council on Animal Care [23]. Eight lactating multiparous Holstein cows fitted with ruminal cannulas averaging 686±35 kg of body weight and 112±21 days in milk were assigned to four treatments in a double 4×4 Latin Square design with four 21-d periods (14-d adaptation and 7-d sampling). The cows were kept in individual stalls and had free access to water. Diets were offered in equal amounts twice daily at 0830 and 1530 h for *ad libitum* intake (10% refusals as served) and milked twice daily in their stalls at 0800 and 1900 h. Cows were fed a total mixed ration (TMR; Table 1) with no FM (control, CON), or diets containing (DM basis) 5% FM, 10% FM (10FM) and 15% FM (15FM). The four total mixed diets were equal in protein and energy of lactation and were formulated to meet nutrient requirements for cows that average 657 kg of body weight and produce 37.7 kg/d of milk with 3.8% of fat, according to NRC [24].

**Table 1:** Ingredients and chemical composition of experimental diets.

	Control*	5FM*	10FM*	15FM*
Ingredients, % of dry matter (DM)				
Corn silage	29.22	29.08	28.98	29.06
Grass silage	31.53	31.57	31.75	31.39
Ground corn	21.13	20.23	19.16	18.90
Soya meal	10.76	7.45	4.14	2.04
Top Suplement†	1.74	1.74	1.79	0.85
Beet pulp	3.43	2.96	2.50	1.61
Calcium carbonate	0.55	0.55	0.54	0.58
Mineral and vitamins‡	1.64	1.63	1.63	1.53
Flax meal	0	4.79	9.53	14.06
Chemical analysis¶				
DM, %	37.7±1.60	37.9±1.60	37.6±1.60	38.1±1.60
Crude protein, % of DM	17.0±0.15	17.4±0.15	17.6±0.15	17.9±0.15
Acid detergent fiber, % of DM	18.3±0.34	18.5±0.34	19.2±0.34	19.3±0.34
Neutral detergent fiber, % of DM	28.4±0.31	28.6±0.31	29.5±0.31	29.6±0.31
Ether extract, % of DM	2.4±0.07	2.4±0.07	2.4±0.07	2.4±0.07

*Control diet with no flax meal (FM) or a diet with 5%, 10% and 15% FM (DM basis).

† Contained 20% of canola meal, 30% of corn gluten meal, 20% of soybean meal, and 30% of brewer's corn.

‡ Contained 9.2% Ca; 4.79% P; 4.78% Mg; 1.52% S; 13.72% Na; 1.37% K; 19.5 mg/kg Se; 23 mg/kg I; 2013 mg/kg Fe; 1068 mg/kg Cu; 1796 mg/kg Mn; 2657 mg/kg Zn; 57 mg/kg Co; 265 mg/kg Fl; 442000 UI/kg vitamin A; 56670 UI/kg vitamin D; and 2630 UI/kg vitamin E.

¶ Values with standard errors of the mean.

### Sampling and preparation

On Day 21 of each period, ruminal contents were collected 0, 2, 4 and 6 h after the morning meal from different locations within the rumen (the cranial dorsal, cranial anterior ventral, medium ventral, caudal dorsal and caudal ventral locations) to obtain a representative sample. The ruminal contents were strained through four layers of cheesecloth. One portion was kept at −20°C and freeze-dried for EL analysis. Another portion of 1L of strained ruminal fluid was taken 2 h post-feeding for microbial isolation as described by Lee et al. [25]. The resultant microbial pellets were freeze-dried, ground with a mortar and frozen at −80°C for molecular analysis.

### Enterolactone analysis

EL analysis of rumen samples obtained from the animal trial was performed using an EIA kit (Cayman Chemical, Ann Arbor, MI, USA). The detailed procedures of extraction and analysis were described by Gagnon et al. [18]. Ruminal samples for the three post-feeding times (2, 4 and 6 h) were pooled within cow and period as previously carried out by Gagnon et al. [18] to obtain only one composite sample for EL analysis.

### DNA extraction

DNA was extracted from approximately 10 mg of freeze-dried rumen samples using the FastDNA Spin Kit for Soil (QBiogene, Cambridge, UK) following the manufacturer's guidelines, although 3×30S bead beating with 1 min intervals on ice was employed. The quality and quantity of DNA were determined using a spectrophotometer (Nanodrop ND-1000, Thermo Fisher Scientific, Wilmington, DE, USA) (260 and 280 nm).

### PCR-T-RFLP analysis of the total bacterial population

PCR T-RFLP was conducted as described by Huws et al. [26], [27] in triplicate with subsequent pooling and use of the restriction enzymes Hae III or Msp I (Promega, Madison, USA). Restriction digested were run on a ABI3130xl DNA sequencer (Applied Biosystems, CA, USA) and T-RFs checked and exported using Genemapper software (Applied Biosystems, CA, USA). Peaks <0.5% of the cumulative peak height were removed [28]. Data were imported into Bio-Rad fingerprinting (Bio-Rad, Hertfordshire, UK) and clustering analysis was undertaken using separation criteria based on a 0.5 bp size difference in peaks and the Pearson's coefficient.

### PCR-DGGE analysis of the total bacterial population

V6–V8 16S rRNA PCR was performed as described by Huws et al. [26], [29], [30] and Kim et al. [31]. Amplicons were loaded onto 6% polyacrylamide gels with a 35–60% denaturing parallel gradient and the electrophoresis performed in a D-Code system (Bio-Rad Laboratories; [31]), running for 10 min at 200 V and then for 16 h at 85 V and 60°C. Gels were then stained with silver nitrate [32], scanned using a GS-710 calibrated imaging densitometer (Bio-Rad, Hemel Hempstead, UK) and the saved image imported into the software package Fingerprinting (Bio-Rad) for analysis [26], [31]. UPGMA dendrograms were constructed using the Dice coefficients and a position tolerance of 0.5% and optimization parameter of 1%. The band number was calculated using generated binary data. DNA extracted from bands of interest were cut, re-amplified and cloned for subsequent sequencing using pGEM-T easy vector system (Promega, Southampton, UK). Clones obtained were sequenced using an ABI3130xl DNA sequencer (Applied Biosystems, CA, USA). These sequences have been submitted to the DDBJ/EMBL/GenBank databases under accession numbers HQ849553-567. Sequences were compared with deposited sequences within Blast (http://blast.ncbi.nlm.nih.gov/Blast.cgi) and the ribosomal database project (RDP – II Release 10; [33]; http://rdp.cme.msu.edu), which gives taxonomic information.

### Quantitative PCR

To investigate total bacteria DNA concentration, 16S rRNA was quantified within ruminal digesta in triplicate according to Kim et al. [31] and Huws et al. [26], [27], [30].

### *In vitro* incubations and HPLC analysis

In order to check whether the bacteria indicated as having a role in lignan metabolism *in situ* were actually capable of these biochemical conversions, we set up pure culture *in vitro* studies. Frozen-stocks of *Butyrivibrio fibrosolvens* JW 11, *Butyrivibrio proteoclasticus* B316, *Eubacterium ruminantium* 2388, *Fibrobacter succinogens* S85, *Peptostreptococcus anaerobius* 27337, *Prevotella albensis* M 384, *Prevotella brevis* GA33, *Prevotella bryantii* B14, *Prevotella ruminicola* ATCC 19189, *Ruminococcus albus* SY3, and *Ruminococcus flavefaciens* 007 were obtained from the bacterial culture collection of the Institute of Biological, Environmental and Rural Sciences, Aberystwyth University. Pure cultures were grown anaerobically at 39°C for 24 h in Hobson's Medium M2 [34], where 1 ml of each pure culture was added into 9 ml of broth within hungate tubes.

Firstly, stock solutions were prepared: SDG (1.45 nM) in water; SECO (2.75 nM), ED (3.30 nM) and EL (3.35 nM) in methanol. SDG, SECO, ED, EL and β-glucuronidase were obtained from Sigma-Aldrich Company Ltd (Dorset, United Kingdom). To test for the conversion of SDG into SECO, 25 µl of SDG stock solution, 5 µl of β-glucuronidase and 870 µl of Hobson's Medium before addition of pure culture (100 µl) were placed into sterile hungate tubes and purged with CO_2_ before closing. Experiments were conducted in duplicate and negative controls with bacteria but without SDG and positive controls with SDG without any bacteria were also analyzed. To test the conversion of ED into EL, 15 µl of ED stock solution were added to 885 µl of Hobson's Medium. All other procedures were as described for conversion of SDG into SECO. As a further positive control samples containing 100 µl of sieved rumen fluid instead of any pure bacterial culture were added with SDG, SECO or ED respectively, again in duplicate. The rumen fluid for the *in vitro* work was obtained from 3 cannulated cows housed at IBERS Abersytwyth University and subsequently pooled for the experiment. All animal experiments are conducted under the authorities of the UK Animal (Scientific Procedures) Act (1986). The tubes were immediately incubated for 24 h at 39°C. Subsequently, incubations were centrifuge (1 min, 13.000 *g*). The supernatant was partially purified on Sep-Pak C_18_ cartridges (500 mg; Waters Ltd. United Kingdom) following the manufacturer's instructions. Samples were then dried down at 80°C under vacuum and resuspended in 200 µl of methanol. The presence of lignans was detected by reverse-phase HPLC on a Waters system with a 996 Photodiode Array Detector (PDA; Waters Ltd., United Kingdom) and a Waters C18 Nova-Pak radial compression column (4 µm, 8 mm×100 mm) equilibrated with 95% solvent A (5% acetic acid) at a flow rate of 2 ml.min^−1^. The sample injection volume was typically 40 µl, and compounds were eluted by linear gradient to 70% solvent B (100% methanol) over 15 min and monitored from 240 to 400 nm. The concentration of each lignan was quantified using pure standards.

### Statistical Analysis

**Figure 2:**
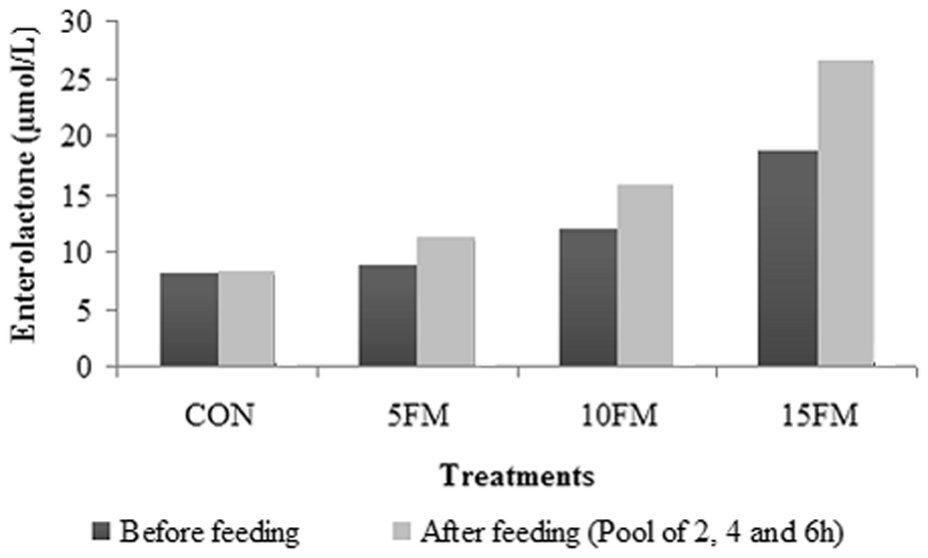
Concentration of enterolactone (µmol/L) in ruminal fluid of Holstein cows fed flax meal (FM). Cows were fed a control diet (CON) or a diet with 5% FM (5FM), 10% FM (10FM) and 15% FM (15FM). There was a linear effect of treatment (*P*<0.0001) before feeding and in the post-feeding pool of ruminal fluid. The standard error was 0.001 for all sampling times.

Band and peak numbers from TRFLP, Q-PCR and EL concentration were analyzed using the MIXED procedure of SAS (2000, SAS Institute, Cary, NC, USA). Treatment 5% FM was excluded for molecular bacterial analysis (PCR-DGGE, PCR-T-RFLP and Q-PCR) as the main objective was to compare the microbiota present under the control treatment *versus* high inclusions of FM in the diet (10% and 15%). Therefore, for the statistical analysis of DGGE band numbers, the number of T-RFLP peaks, and Q-PCR, the statistical model was a double incomplete 4×4 Latin square design while a double complete 4×4 Latin square was considered for EL concentration with the general model:
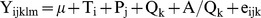
Where Y_ijklm_ = the response variable, μ = overall mean, T_i_ = global effect of treatment (i = CON, 5FM, 10FM and 15FM), P_j_ = the fixed effect of period (j = 1 to 4), Q_k_ = fixed effect of square (k = 1, 2), A/Q_lk_ = random effect of cow within square, and e_ijklm_ = residual error. Enterolactone data were transformed (log) as performed by Nesbitt et al. [35] due to the lack of variance homogeneity and variation in its concentration. However the results in the Fig. 2 were expressed on the original scale of measurements. When a tendency was observed for an interaction (*P*≥0.10) between treatment and time, the effect of treatment was examined within each time group, and then the treatment effects were compared at the relevant time. Normality and homogeneity were analyzed with the procedure UNIVARIATE of SAS (2000, SAS Institute, Cary, NC, USA). Statistical differences were declared at *P*<0.10.

## Results

### Diet composition

Flax meal was fed at 4.79, 9.53 and 14.06% of dry matter, respectively, for treatments with 5, 10, and 15% of FM (Table 1). Concentrations of crude protein, acid-detergent fibre, neutral-detergent fibre and ether extract were similar among diets.

### Ruminal concentration of EL

Flax meal supplementation increased concentration of EL linearly (*P*<0.0001) (Fig. 2) before feeding and in the pool of post-feeding times. However, there was an interaction (*P* = 0.1055) between diet and sampling time for EL concentration in ruminal fluid as a result of a greater increase between before and after feeding for cows fed 15% FM compared to those fed the other diets.

### Bacterial 16S rRNA quantity and diversity

Total bacterial 16S rRNA concentrations averaged 6.46, 7.65, and 7.27 ng.g^−1^ 16S rRNA (S.E. = 0.41) for treatments CON, 10FM, and 15FM, respectively, and they were similar (*P* = 0.1505) among treatments.

**Figure 3:**
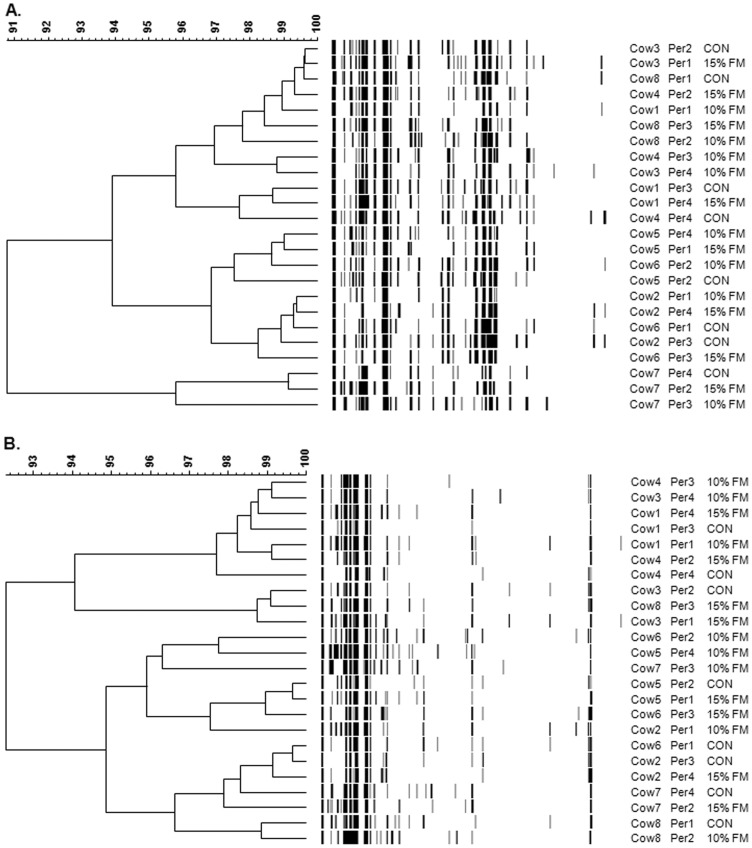
PCR-T-RFLP-derived unweighted pair group method with arithmetic mean (UPGMA) dendograms showing the effect of flax meal inclusion on the rumen microbiota. The T-RFLP was based on Hae III (A) and MSP I (B) restriction enzymes. Cows were fed a control diet (CON) or a diet with 10% (10% FM) and 15% flax meal (15% FM). Scale relates to percent similarity and data are presented per period (Per).

T-RFLP-derived unweighted pair group method with arithmetic mean (UPGMA) dendrograms did not show any global clustering dependent on diet for neither Hae III nor MSP I (Fig. 3A and 3B) potentially due to the fact that basal animal variation was high. A higher number of peaks for restriction enzyme MSP I was observed for cows supplemented with FM, but no treatment effect was observed with respect to the number of peaks obtained from Hae III (Table 2).

**Table 2:** Mean peak/band number following HaeIII- and MSP1-based 16S rRNA T-RFLP and V6–V8 PCR-DGGE of rumen bacteria within rumen samples obtained from Holstein cows fed a control diet with no flax meal (CON), 10% (10FM) and 15% (15FM) of flax meal (FM) in the dry matter.

	Treatments			S.E.M.	*P*-value
	CON	10FM	15FM		
Hae III	101.75	94.12	97.12	4.26	0.5222
MSP I	75.62	99.62	82.25	3.79	0.0027
PCR-DGGE	46.87	42.25	40.75	2.4	0.2149

**Figure 4:**
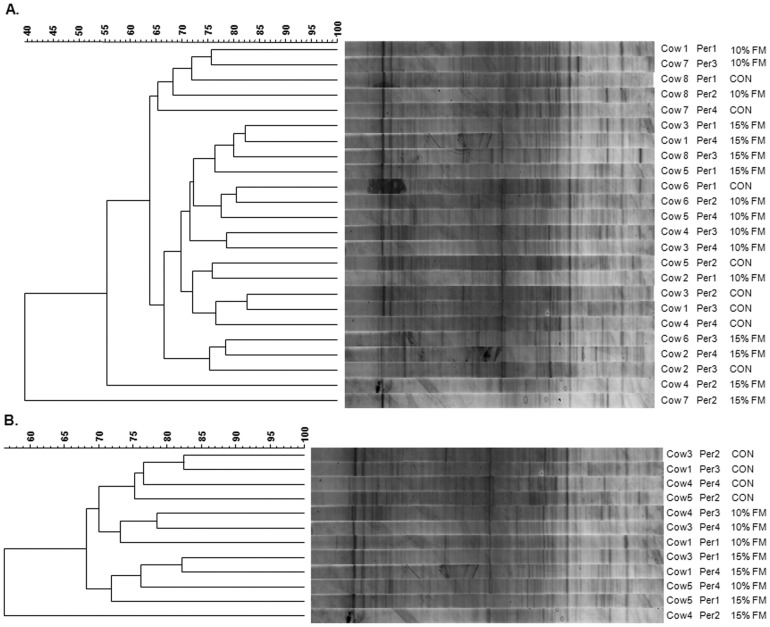
PCR-DGGE-derived unweighted pair group method with arithmetic mean (UPGMA) dendograms showing the effect of flax meal inclusion on the rumen microbiota. The 16S rRNA PCR-DGGE is shown for all cows (A) and within four cows (B) which clustered based on the diet. Cows were fed a control diet (CON) or a diet with 10% (10% FM) and 15% flax meal (15% FM). Scale relates to percent similarity and data are presented per period (Per).

**Figure 5:**
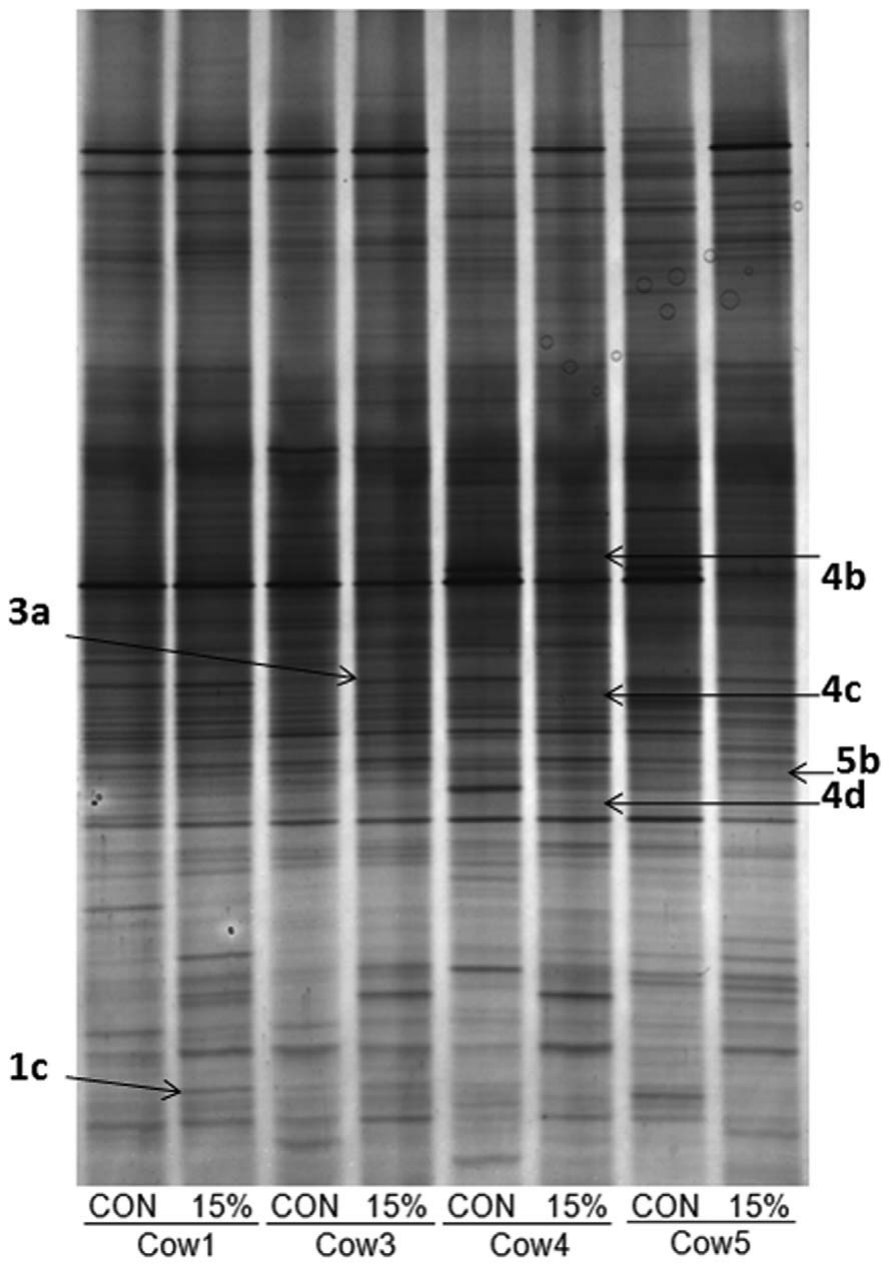
DGGE gel for sequencing purpose. This DGGE gel shows rumen bacterial diversity within cows fed no flax meal (CON) or 15% flax meal (15%) in the diet (dry matter basis). Arrows show bands that appeared upon flax meal supplementation, which were cut and sequenced.

DGGE was employed as often data obtained using both techniques differ and also it is easier to subsequently sequence DGGE bands. When all cows were considered, there was no clear clustering by treatment likely due to the individual variation among animals as also noted in the T-RFLP analysis (Fig. 4A). However, when each cow was observed individually, four animals clustered closely based on diets (approx. 68% similarity seen between bacterial diversity present on the CON and 15FM diets; Fig. 4B), suggesting a treatment effect on the bacterial population (Fig. 5). These animals had an initial diversity which was far more similar compared with the other cows on trial. Thus, in order to investigate and identify which bacteria may be involved in SDG conversion and consequently EL production, the DNA extracted from bands present when the FM diet (15%) was fed compared to the CON diet were cut and sequenced on an individual animal basis for these 4 animals. Sequences revealed that bacteria potentially associated to the metabolism of lignans belonged to uncultured bacteria phylogenetically classified as *Prevotella*, *Succinivibrionaceae*, *Alphaproteobacteria* and uncultured rumen bacteria *Succinivibrio*, *Lachnospiraceae*, *Bacteroidales*, *Anaerovorax* and *Prevotella*, and strain of *Fibrobacter succinogenes* (Table 3).

**Table 3:** Taxonomic identification of DGGE bands potentially associated with enterolactone production in ruminal fluid.

Band position (clone no.)	Nearest match (accession number; Maximum% sequence similarity)	Ribosomal Database Project Classification
Cow 1 (band position A clone 1)	Uncultured bacterium isolate 16S ribosomal RNA gene (EU624093.1; 99%)	unclassified_*Succinivibrionaceae*
Cow 3 (band position A clone 1)	Uncultured rumen bacterium clone CF23 16S ribosomal RNA gene (EU871348.1; 94%)	unclassified_“*Lachnospiraceae*”
Cow 3 (band position A clone 2)	Uncultured bacterium clone p-1030-a5 16S ribosomal RNA gene (AF371866; 96%)	unclassified_*Alphaproteobacteria*
Cow 4 (band position A clone 1)	Uncultured rumen bacterium clone YRC13 16S ribosomal RNA gene (EU259389.1; 98%)	unclassified_“*Bacteroidales*”
Cow 4 (band position B clone 1)	Uncultured rumen bacterium clone BF399 16S ribosomal RNA gene (EU850583.1; 96%)	genus *Anaerovorax*
Cow 4 (band position B clone 2)	Uncultured rumen bacterium 5C3d-4 gene for 16S rRNA (AB034106.1; 99%)	genus *Prevotella*
Cow 4 (band position B clone 3)	Uncultured rumen bacterium clone P5_D21 16S ribosomal RNA gene (EU381799.1; 98%)	genus *Succinivibrio*
Cow 4 (band position B clone 4)	*Fibrobacter succinogenes* strain H23 16S ribosomal RNA gene (JF970205.1; 99%)	genus *Fibrobacter*
Cow 4 (band position C clone 1)	Uncultured rumen bacterium clone TWBRB64 16S ribosomal RNA gene, partial sequence (FJ028779.1; 94%)	unclassified_“*Prevotellaceae*”
Cow 4 (band position C clone 2)	Uncultured rumen bacterium clone BE5 16S ribosomal RNA gene (AY244922.1; 96%)	genus *Prevotella*
Cow 4 (band position C clone 3)	Uncultured bacterium clone NED5F11 16S ribosomal RNA gene (EF445279.1; 99%)	genus *Prevotella*
Cow 4 (band position C clone 4)	Uncultured rumen bacterium clone CTRS1H03 16S ribosomal RNA gene (GQ327793.1; 99%)	genus *Prevotella*
Cow 5 (band position A clone 1)	Uncultured rumen bacterium clone CTRS1H03 16S ribosomal RNA gene (GQ327793.1; 97%)	genus *Prevotella*

There was no treatment effect (*P* = 0.21) on band numbers from V6–V8 16S rRNA PCR-DGGE of bacteria obtained from ruminal samples of cows receiving either the CON, 10FM or 15FM diets (Table 2).

### *In vitro* experiment

Based on the *in situ* data, culturable bacteria belonging to the same genera found with DGGE were chosen to further probe the ability of these bacteria to metabolize SDG. In addition, species available in IBERS collection of the genus Butyrivibrio sp., *Eubacterium* sp. *and Ruminococcus* sp. were also selected for our *in vitro* experiments as they are known to play a role in the metabolism of lignans in humans [10], [13], [14] and goats [17]. After anaerobic incubation with SDG, we found using HPLC that all of the 11 bacteria investigated were able to hydrolyze the sugar portions of SDG and release SECO, namely the deglycosylation reaction [13]. The HPLC results were converted into molarities (SDG *M* = 686.7 g/mol; SECO *M* = 362.4 g/mol; ED *M* = 302.36 g/mol), and expressed as the percentage of remaining SDG after 24 h of incubation and as SECO production after 24 h of incubation in relation to the initial concentration of SDG of 1.14 m*M* (blank without incubation). Each bacterium presented different efficiencies in ability to convert SDG to SECO, with *Prevotella* spp. being the most efficient and *B. fibrosolvens*, *P. anaerobius* and *F. succinogenes* having similar capacities to convert SDG into SECO (Table 4).

**Table 4:** Efficiency of conversion of SDG into SECO by selected pure cultures of ruminal bacteria and conversion of SDG into SECO and ED using ruminal fluid as inoculum, assessed using *in vitro* cultures and HPLC^1^.

	% SDG remaining after 24 h incubation	% of SECO produced based on initial SDG amount	% of ED produced based on initial SDG amount
*Prevotella bryantii*	2.1	81.7	n.d.
*Prevotella albensi*	2.1	60.4	n.d.
*Prevotella ruminicola*	4.8	56.5	n.d.
*Prevotella brevis*	44.9	49.4	n.d.
*Peptostreptococcus anaerobius*	20.5	49.2	n.d.
*Butyrivibrio fibrosolvens*	26.8	50.8	n.d.
*Fibrobacter succinogens*	33.3	39.1	n.d.
*Ruminococcus albus*	60.6	14.9	n.d.
*Eubacterium ruminantium*	79.1	11.8	n.d.
*Butyrivibrio proteoclasticus*	39.9	6.8	n.d.
*Ruminococcus flavefaciens*	76.9	3.3	n.d.
Ruminal fluid	2.1	46.6	8.0

1 The HPLC results were converted into molarities (SDG *M* = 686.7 g/mol; SECO *M* = 362.4 g/mol; ED *M* = 302.36 g/mol), and expressed as the percentage, in relation to the initial concentration of SDG of 1.14 m*M*; n.d.: non-detected.

Neither ED nor EL was observed on the HPLC chromatograms after 24 h of incubation when the pure cultures were added with SDG as substrate. In addition, when ED was added as substrate, none of the 11 studied bacteria were able to convert ED into EL, and only ED peaks were identified on HLPC chromatograms after 24 h incubation (data not shown). In the positive control samples (SDG or ED incubated without any bacteria) SDG and ED concentration did not alter after incubation. The negative control samples (bacteria without any substrate) did not show any presence of lignans after incubation. However, when ruminal fluid was used as inoculum, positive results were observed, as for conversion of SDG into SECO and ED (Table 4); and for conversion of SECO into ED and conversion of ED into EL (data no shown).

## Discussion

The study was designed to identify the ruminal bacteria responsible for the conversion of plant lignans into mammalian lignans. This study indicates that many bacteria, in particular the *Prevotella* spp. genus, may be important for converting plant into mammalian lignans in the rumen. Previous results have demonstrated that the main site of mammalian lignan formation in ruminant animals was the rumen [18]. Indeed, increased levels of the mammalian lignan EL in ruminal fluid of cows supplemented with flax hulls [7], [36] and sheep infused with purified SDG in the rumen [17] have been reported earlier. Feed ingredients such as soy and corn contain lignans [37], [38] also lead to EL production [39], which may explain why EL was present in the rumen of cows fed CON. However, FM was clearly the main source of lignans (i.e., SDG) in the diet as it is known to be one of the richest sources of plant lignans [39].

T-RFLP indicated no overall change in the bacterial communities with FM supplementation for both Hae III and MSP1 endonucleases, even when animals were compared separately due to the basal variation in the rumen microbiota highlighted by T-RFLP. Nonetheless, overall changes were observed in the ruminal microbiota upon FM feeding when the PCR-DGGE technique was applied when cows were analyzed separately. Indeed, four animals clustered closely based on diets and distinct bands appeared when FM was supplemented. Discrepancies between T-RFLP and DGGE data have previously been noted, which may be because a different amplicon is used for both techniques [40]–[42]. For the purpose of investigating and identifying which bacteria may be involved in SDG metabolism, the DNA extracted from bands of interest present when the 15% FM diet was fed compared to the CON diet were cut and sequenced on an individual animal basis. The sequencing data obtained indicated which rumen bacteria could potentially be responsible for metabolizing the flax lignans. The subsequent *in vitro* incubations and HPLC analysis confirmed that the 11 ruminal species of bacteria selected based on our data and those of monogastric trials were able to convert SDG into SECO, which is a deglycosylation reaction catalyzed by the enzyme β-glucuronidase [43]. In the present experiment, although β-glucuronidase had been added to SDG to allow the deglycosylation reaction to occur during the 24 h incubation, each strain possessed different capacities to metabolize SDG into SECO. Bacteria from genera *Prevotella* presented a higher efficiency of conversion of SDG into SECO, followed by *B. fibrosolvens* and *P. anaerobius*. Conversely, *R. albus*, *E. ruminantium* and *R. flavefaciens* were less efficient in SDG conversion compared with *Prevotella* spp. *B. proteoclasticus* also showed low SECO production and the lowest recovery of both plant lignans (SDG and SECO), suggesting that intermediate compounds other than ED and EL were obtained during the metabolism of plant SDG. Indeed, recovery of the substrate and the metabolites SECO, ED and EL investigated in this experiment did not reach 100%. Wang et al. [11] identified seven metabolites of SDG when incubating SDG with human faeces. For example, matairesinol is one compound produced from SECO metabolism as suggested by Heinonen *et al.* [44]. Moreover, Landete et al. [45] described an intermediate metabolite identified as 2,3-bis(3,4-dihydroxybenzyl)butane-1,4 diol that is formed after the demethylation of SECO, which is further dehydroxylated into ED. Likewise, *in vitro* incubation of pure SDG may lead to the formation of intermediate metabolites as suggested by some unidentified peaks observed on chromatograms, although these compounds were not looked at in the present experiment.

The genus *Bacteroides*, which belongs to the order *Bacteroidales*, was identified by Clavel et al. [8] as one of the human intestinal bacteria responsible for the catalytic conversion of SDG to SECO via deglycosylation (β-glycosidases). The family *Prevotellaceae* and the genus *Prevotella* also belong to the order *Bacteroidales*. As the genus *Prevotella* within the family *Prevotellaceae* represented more than 45% of the identified bands in the current study and were the main SECO producers *in vitro*, this may suggest that they play an important role in lignan conversion within the rumen. *F. succinogenes* is a fibrolytic bacterium and its enzymes possess endoglucanases, cellobiosidase, cellodextrinase, xylanases, and β-glucosidase activities [46]. In the present experiment, *F. succinogenes* S85 was able to convert SDG into SECO. This agrees with the activity of β-glucosidase that has been demonstrated by *F. succinogenes* S85 [46]. Indeed, β-glucosidase is the enzyme required for the conversion of SDG to SECO [14]. In a recent study, Zhou et al. [17] found that *Ruminococcus gnavus* was potentially responsible for the conversion of plant lignans into EL in goats. In the current study, it was shown that *R. albus* and *R. flavefaciens* also were involved in the conversion of SDG to SECO.

In the present experiment, none of the 11 bacteria were able to produce EL from SDG or ED. Furthermore, the EL concentration was below detection level in ruminal fluid used for the *in vitro* cultures, this may be because grass fed to cows was poor in SDG and therefore the bacteria used in the *in vitro* trials were not adapted to convert SDG. This agrees with the results of Antignac et al. [47] who observed a large variation in milk EL concentration as a response to animal feeding. In addition, Höjer et al. [48] reported also large variations in equol concentration between individual cows, indicating the possibility of selecting high or low producers of equol. Similar results have been observed by Petit et al. (unpublished data) for high and low producers of EL. The lack of *in vitro* production of EL from SDG also could indicate that bacteria metabolizing ED into EL did not grow well under the present experimental conditions. Indeed, previous results have shown no conversion [11] or conversion [49], [50] of ED into EL after *in vitro* incubation of SDG with a rat faecal suspension. In addition, Borriello et al. [51] reported that there was no conversion of ED into EL when no viable bacteria were presented in diluted human faeces used as inoculum. Thus, the differences in outcome of ED conversion to EL in these published experiments are likely linked to presence of lower colonic bacteria [52]. Conversely, previous results from Côrtes et al. [7] have shown that EL formation occurs when ruminal fluid from cows fed flax products (hulls and seeds) was used as inoculum. Discrepancies between studies could also be due to other factors such as differences in the physical form of the substrate. Indeed, purified SDG was added in the present trial and not flax meal.

In summary, inclusion of FM increased concentration of the mammalian lignan EL in the rumen and altered the ruminal microbiota as demonstrated using DGGE. Sequencing of key bands present upon inclusion of 15% FM in the diet and absent when no FM was fed showed that diverse rumen bacterial taxa may play a role in the metabolism of flax lignans. Subsequent *in vitro* studies supported the *in situ* data and showed that *Prevotella* spp. in particular may contribute to the conversion of SDG into SECO in the rumen. However, further studies are required to identify ruminal bacteria responsible for the formation of ED and EL. Identification and characterization of the enzymes involved in the conversion of plant into mammalian lignans, which are linked to better human health, is also paramount for developing animal products enriched in enterolactone.
